# The Transcription Factor Basic Pentacysteine 5, RsBPC5, Enhances Lead Stress Tolerance in *Raphanus sativus*

**DOI:** 10.3390/plants14152362

**Published:** 2025-08-01

**Authors:** Jian Xiao, Yongli Wen, Wenjing Kang, Fangzhou Yu, Chuan Liu, Zhenyu Peng, Dianheng Xu

**Affiliations:** 1 Jiangsu Provincial University Key Laboratory of Agricultural and Ecological Meteorology, Key Laboratory of Ecosystem Carbon Source and Sink-China Meteorological Administration (ECSS-CMA), School of Ecology and Applied Meteorology, Nanjing University of Information Science & Technology, Nanjing 210044, China; 202413020032@nuist.edu.cn (F.Y.); liuchuan55@outlook.com (C.L.); zhengyupeng123@outlook.com (Z.P.); dianhengxu@outlook.com (D.X.); 2 Institute of Loess Plateau, Shanxi University, Taiyuan 030031, China; ylwen@sxu.edu.cn; 3 Jiangsu Provincial Key Lab for Organic Solid Waste Utilization, Nanjing Agricultural University, Nanjing 210095, China; 2017203060@njau.edu.cn

**Keywords:** *Raphanus sativus*, lead stress, BPC, antioxidant defense

## Abstract

Radish (*Raphanus sativus*), a commonly grown root vegetable prized for its nutrition and culinary use, is particularly vulnerable to lead (Pb) stress, which mainly results in Pb accumulation in the roots. However, the molecular mechanisms underlying Pb accumulation in radish remain largely unknown. In this study, we investigated the role of BASIC PENTACYSTEINE (BPC) genes in radish’s response to Pb stress. Phylogenetic analysis revealed that radish contains 10 BPC genes, which are distinctly clustered in Cluster III. Expression analysis revealed that, except for *RsBPC2*, *RsBPC4*, and *RsBPC7*, the expression of most *RsBPC* genes was significantly altered under Pb stress. Notably, the expression of *RsBPC5* gradually decreased with prolonged Pb exposure. Subcellular localization analysis confirmed that RsBPC5 is localized in the nucleus and acts as a transcriptional repressor. Functional assays demonstrated that transient overexpression of *RsBPC5* enhanced the tolerance of radish plants to Pb stress via reducing Pb accumulation and activating the antioxidant defense system. Collectively, our findings suggest that RsBPC5 plays a key role in radish’s response to Pb stress, potentially improving Pb tolerance by modulating Pb uptake and strengthening antioxidant defense mechanisms.

## 1. Introduction

Lead (Pb) is one of the most toxic heavy metal pollutants, posing a significant threat to living organisms [1,2]. Once absorbed, Pb severely impairs growth and development. Furthermore, no biological function for Pb has been identified in organisms. Moreover, due to its inability to undergo thermal decomposition, biodegradation, or metabolic transformation, lead (Pb) is particularly difficult to remediate once released into the environment [3]. Plants primarily absorb Pb through their roots. Excessive Pb accumulation adversely affects plant morphology, growth, and photosynthesis by inhibiting enzyme activities, inducing dehydration, altering cell membrane permeability, and disrupting the uptake of essential nutrients. At the cellular level, Pb toxicity suppresses enzyme functions and exacerbates oxidative stress by promoting excessive generation of reactive oxygen species (ROS) [4]. To cope with Pb stress, plants have evolved various defense mechanisms [5]. These mechanisms typically sense and transmit Pb stress signals from the cell membrane to the cytoplasm and nucleus, thereby inhibiting gene expression reprogramming and biochemical cascade adjustments. In plants, several transcription factor families, including MYB, bZIP, and others, have been validated as vital regulators in responses to Pb stress [6,7,8,9].

BASIC PENTACYSTEINE (BPC) proteins are a class of plant-specific transcription factors widely distributed among terrestrial plants [10,11]. In *Arabidopsis thaliana*, seven BPC genes (AtBPC1–AtBPC7) have been identified. Based on domain architecture and sequence similarity, these genes are classified into three groups: Class I (AtBPC1–AtBPC3), Class II (AtBPC4–AtBPC6), and Class III (AtBPC7) [12]. AtBPC5 is considered a pseudogene, likely encoding a non-functional protein due to a frameshift-induced premature stop codon. All six functional AtBPC proteins share a conserved C-terminal DNA-binding domain that specifically recognizes and binds to GAGA-rich promoter motifs in target genes, thereby modulating their transcription [11]. Functionally, BPC transcription factors regulate ovule development, leaf development, and root formation [13,14,15]. For instance, AtBPC1 directly represses *INNER NO OUTER*, *SEEDSTICK*, and *FUSCA3*, negatively impacting ovule and embryo development [13,16]. Class I members (AtBPC1, AtBPC2, AtBPC4, and AtBPC6) collectively suppress *ABSCISIC ACID INSENSITIVE4*, regulating root development [14]. In apple, MdBPC2 controls plant stature by regulating auxin biosynthesis genes *MdYUC2a* and *MdYUC6b*, promoting dwarfing [17]. Moreover, BPC transcription factors participate in environmental stress responses [18]. In *Arabidopsis*, AtBPC2 reduces seedling tolerance to osmotic stress by repressing *AtLEA4-5* [19], while AtBPC1 and AtBPC2 jointly suppress *GALACTAN SYNTHASE1* to modulate salt stress tolerance [10,20]. Recently, BcBPC9 was found to positively regulate cadmium stress responses in flowering Chinese cabbage, indicating a role for BPCs in heavy metal stress [21]. However, whether BPC proteins are involved in responses to other heavy metals remains unclear.

Radish (*Raphanus sativus*) belongs to the genus Raphanus in the family Cruciferae [22]. It is widely consumed in China and holds an important place in the national diet, primarily due to its long cultivation history and cultural significance. In recent years, the abundant bioactive compounds in radish, such as glucosinolates and flavonoids, have garnered increasing attention for their nutritional and medicinal potential. Moreover, radish plays a crucial role in the vegetable supply chain by contributing to the efficient production, broad distribution, and consistent availability of fresh vegetables to consumers [23,24]. However, radish is highly susceptible to heavy metal contamination, particularly Pb stress [25]. Many studies have shown that Pb predominantly accumulates in the fleshy roots and hypocotyls of radish, accounting for up to 50% of the plant’s total Pb content. This accumulation not only compromises radish yield and quality but also poses a potential risk to human health by entering the food chain [26,27]. Therefore, developing effective strategies to reduce Pb accumulation in radish and elucidating the molecular mechanisms underlying Pb detoxification are crucial for ensuring food safety and promoting sustainable vegetable production.

To date, the BPC gene family has not been characterized in radish. Here, we conducted a comprehensive analysis of the radish BPC gene family and identified a significant reduction in *RsBPC5* expression under Pb stress. We further explored the functional role of RsBPC5 in conferring resistance to Pb stress. Our study provides a foundation for future research into radish responses to heavy metal stress and provides insights for breeding Pb-tolerant radish varieties.

## 2. Results

### 2.1. Identification of BPC Family in R. sativus

To identify the BPC family in *R. sativus*, a Brassicaceae species, the amino acid sequences of seven *Arabidopsis thaliana* BPCs (AtBPC1–AtBPC7) from the TAIR database were used, with *A. thaliana* being a close relative of *R. sativus* within the same Brassicaceae family. These sequences were subjected to BLAST V2.16.0 [28] alignment against the *R. sativus* protein dataset with a significance threshold of *p* < 1 × 10^−5^. Ultimately, 10 candidate RsBPCs in the radish genome were confirmed through further domain analysis using NCBI-CDD with the default Automatic mode and a *p*-value < 0.01.

### 2.2. Phylogenetic Analysis of BPC Family

**Figure 1:**
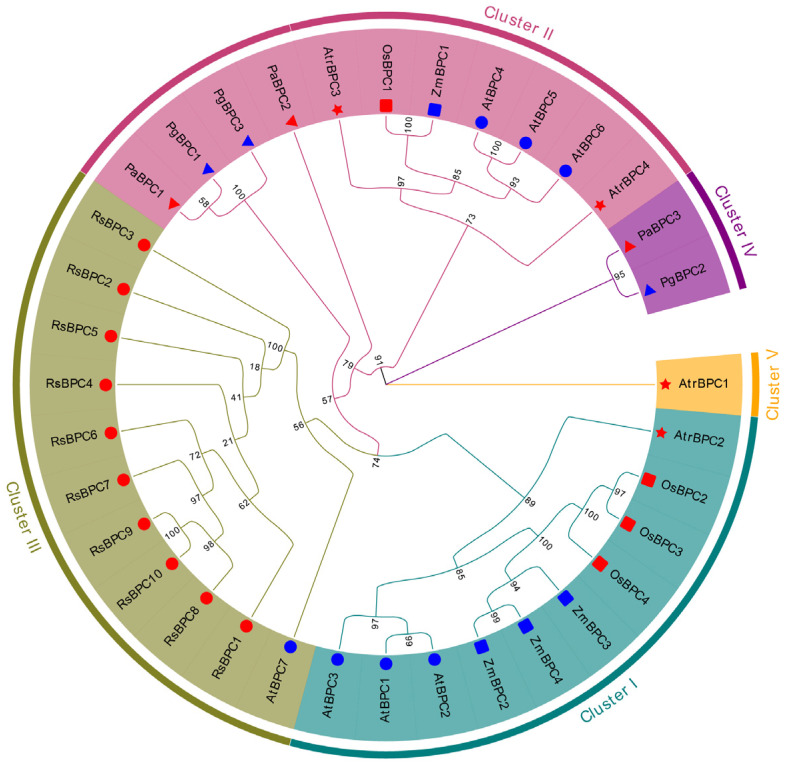
Phylogenetic relationships of BPCs among seed plants. The phylogenetic tree was investigated using IQ-TREE, covering seven species: *Picea abies* (Pa) and *Picea glauca* (Pg) as gymnosperm representatives, *Amborella trichopoda* (Atr) for ANA grade, *Arabidopsis thaliana* (At) and *Raphanus sativus* (Rs) for dicotyledons, and *Oryza sativa* (Os) and *Zea mays* (Zm) for monocotyledons. The symbols used in the tree are as follows: the red circle represents *R. sativus*, the red star represents *A. trichopoda*, the blue circle represents *A. thaliana*, the red triangle represents *P. abies*, the blue triangle represents *P. glauca*, the blue rectangle represents *Z. mays*, and the red rectangle represents *O. sativa*.

To analyze the BPC family in seed plants, a Maximum Likelihood (ML) phylogenetic tree was performed using IQ-TREE for seven selected species. Specifically, *Picea abies* and *Picea glauca* were chosen as representatives of gymnosperms, *Amborella trichopoda* for ANA grade, *A. thaliana* and *R. sativus* for dicotyledons, and *Oryza sativa* and *Zea mays* for monocotyledons. Among the selected plant representatives, a number of BPCs exhibited marked differences. *R. sativus* topped the list with 10 BPCs, and *A. thaliana* had seven. In contrast, *O. sativa*, *Z. mays*, and *A. trichopoda* each had four BPCs. The gymnosperms *Picea abies* and *Picea glauca* had the fewest at three each (Table S1). Further evolutionary analysis divided BPCs into five clusters (Figure 1). Clusters I to III contain a comparable number of BPC members, whereas Cluster IV is markedly smaller. Notably, Cluster II includes BPCs from all examined species except *R. sativus*, whose genes are predominantly located in Cluster III. Also, many BPCs of the monocots *O. sativa* and *Z. mays* are in Cluster I. Meanwhile, some gymnosperm BPCs from *A. trichopoda* are grouped in Cluster IV, while AtrBPC1 from *A. trichopoda* is assigned to Cluster V. The results suggest that the BPC family in seed plants exhibits considerable diversity and species-specific patterns, with significant variation in gene numbers and evolutionary groupings across species.

### 2.3. Protein and Gene Structure Analysis of RsBPCs

**Figure 2:**
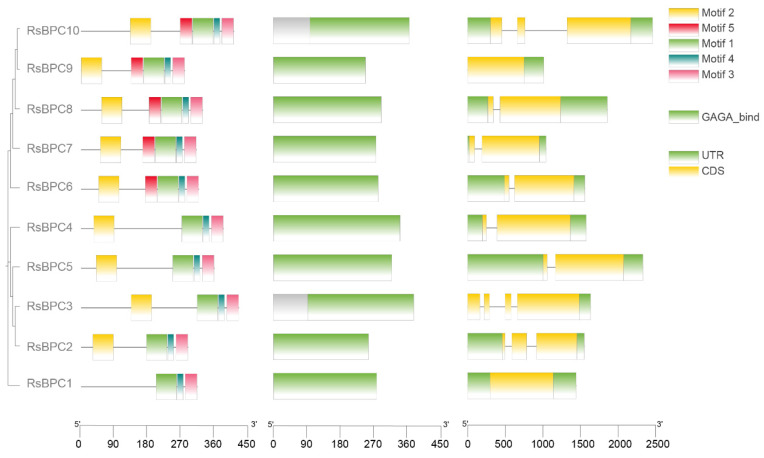
Visualization of protein and gene structure of RsBPCs. Phylogenetic tree was constructed using the Maximum Likelihood (ML) method.

Next, a MEME analysis was conducted on the amino acid sequences of RsBPC proteins. The results revealed that all RsBPC proteins contain motif1, motif3, and motif4, while motif2 is exclusively absent in RsBPC1, and motif5 is only present in RsBPC6 to RsBPC10 (Figure 2). Further analysis using the NCBI-CDD indicates that all amino acid sequences of RsBPCs possess a GAGA_bind domain, which is essential for the function of GAGA transcription factors (Figure 2). Gene structure analysis shows that *RsBPCs* display significant structural diversity, with lengths ranging from 1013 to 2461 base pairs, intron numbers ranging from 0 to 3, and exon numbers ranging from 1 to 4 (Figure 2).

### 2.4. RsBPC5 Was Obviously Downregulated by Pb Stress

**Figure 3:**
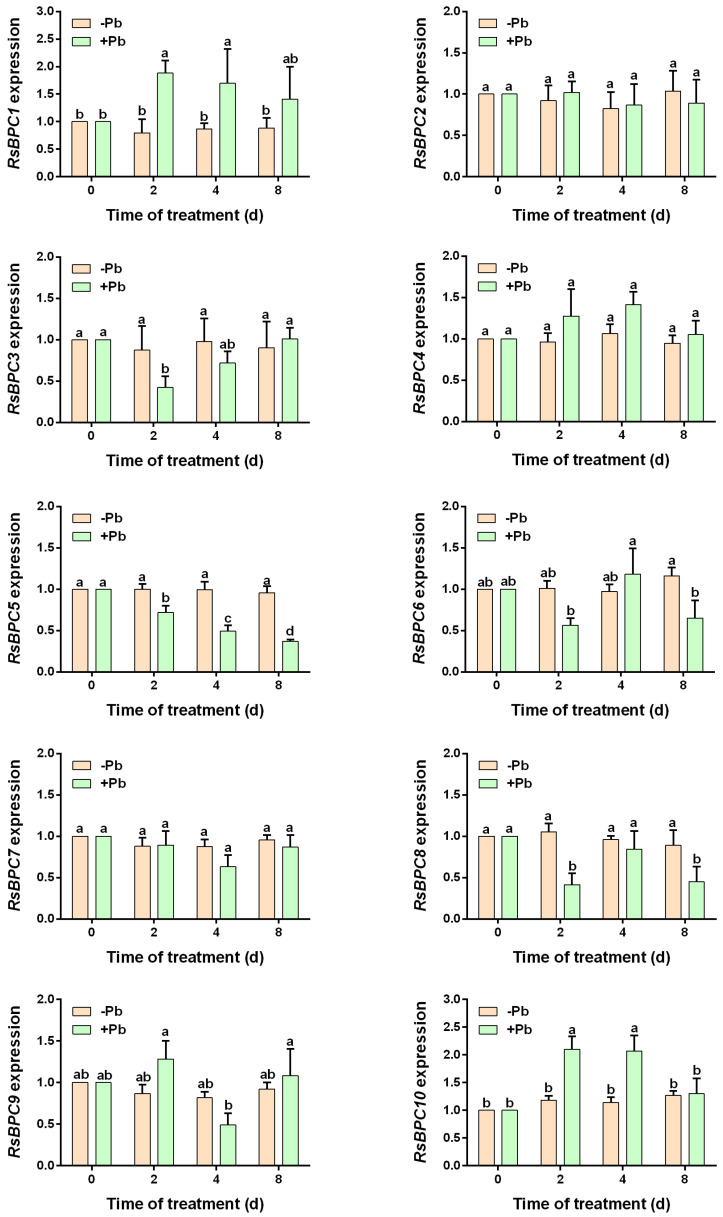
RT-qPCR analysis of *RsBPCs* in radish roots exposed to Pb stress. The expression profiles of *RsBPCs* in radish roots subjected to distinct Pb treatment conditions were analyzed. The expression levels are normalized relative to the 0-day treatment group (−Pb and +Pb groups). Experiment was performed in three independent biological replicates, and error bars represent the standard deviation (SD) calculated from these replicates. Different letters indicate statistically significant differences based on two-way ANOVA and Tukey’s multiple comparisons test (*p* < 0.05).

To explore whether *RsBPCs* in *R. sativus* respond to lead stress, the transcript levels of *RsBPCs* genes in roots subjected to different durations of lead stress were analyzed using RT-qPCR. As shown in Figure 3, the expression of most *RsBPC* members, excluding *RsBPC2*, *RsBPC4*, and *RsBPC7*, was affected to varying degrees under lead stress. Specifically, the expression levels of *RsBPC3*, *RsBPC5*, *RsBPC6*, and *RsBPC9* were downregulated, while *RsBPC1* and *RsBPC10* were upregulated under lead stress. Notably, *RsBPC5* expression showed a negative correlation with Pb treatment duration, exhibiting a clear downward trend as the exposure time increased.

### 2.5. Functional Analysis of RsBPC5

**Figure 4:**
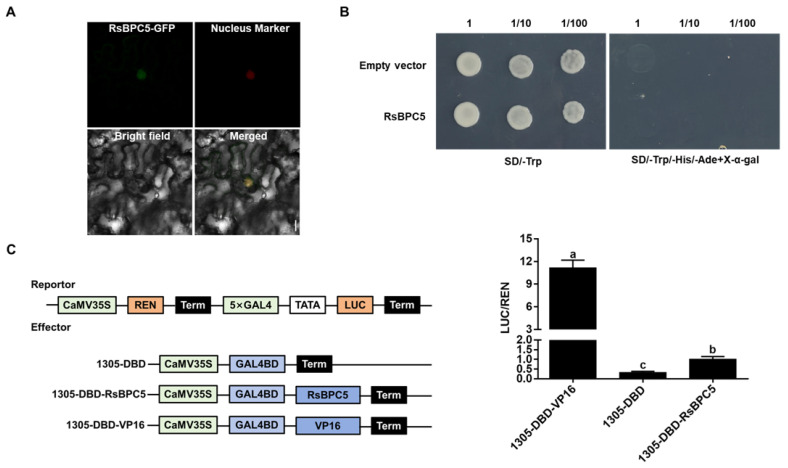
Functional analysis of the radish transcription factor RsBPC5. (**A**) Subcellular localization of RsBPC5. Transcriptional activity of RsBPC5 in yeast (**B**) and tobacco (**C**). VP16, a viral transcriptional activator, was used as a positive control. Experiment in (**A**,**B**) were repeated at least three times with similar results. Experiment in (**C**) was performed in three independent biological replicates, and error bars represent the standard deviation (SD) calculated from these replicates. Different letters in (**C**) indicate statistically significant differences based on one-way ANOVA (*p* < 0.05).

The CDS of *RsBPC5* was fused with *GFP* and co-expressed with a nucleus marker in tobacco leaf epidermal cells. The result showed that RsBPC5-GFP produced a strong fluorescent signal in the nucleus that co-localized with the nucleus marker (Figure 4A), indicating its nuclear localization.

To assess the transcriptional activity of RsBPC5, we used a GAL4-based yeast system to test the transcriptional activation activity of RsBPC5. The coding sequence of *RsBPC5* was fused to the GAL4 DNA-binding domain and transformed into the yeast strain Y2H. The transformants were plated on synthetic dropout (SD/-Trp) medium to select for positive transformants and on SD/-Trp/-His/-Ade/X-α-Gal medium to assess transcriptional activation. If RsBPC5 possessed transcriptional activation activity, the GAL4 DNA-binding domain-RsBPC5 fusion protein would activate the expression of reporter genes (HIS3, ADE2, and MEL1), allowing yeast growth and blue color development on the selection medium. As shown in Figure 4B, yeast-expressing *RsBPC5* failed to grow on the SD/-Trp/-His/-Ade/X-α-Gal medium, indicating that RsBPC5 lacks transcriptional activation activity. To investigate the transcriptional activity of RsBPC5 in vivo, a dual-luciferase reporter system in tobacco was used. The results in Figure 4C revealed a significant increase in the LUC/REN ratio, suggesting that RsBPC5 acts as a transcriptional activator.

### 2.6. RsBPC5 Positively Regulates the Tolerance of Radish to Pb Stress

**Figure 5:**
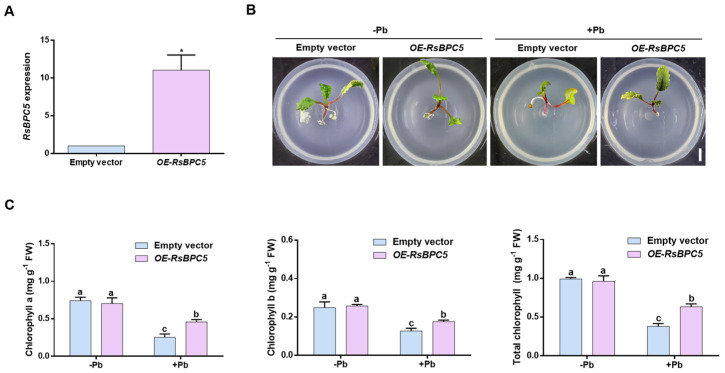
Phenotypic of transient *RsBPC5* overexpression in *Raphanus sativus* under Pb stress. (**A**) Transcript abundance of *RsBPC5* in hairy roots of transient *OE-RsBPC5* seedlings. (**B**) Phenotypic observations of seedlings after 6 days of Pb stress. Scale bar = 1 cm. Each experiment was independently repeated at least three times with six plants per replicate, and the images shown represent one of the biologically replicated seedlings. (**C**) Quantitative analysis of chlorophyll contents in transient *OE-RsBPC5* and empty vector transgenic seedlings after 6 days of Pb stress. Experiments in (**A**,**C**) were performed in three independent biological replicates, and error bars represent the standard deviation (SD) calculated from these replicates. Asterisk in (**A**) indicates statistically significant differences as determined by Student’s *t*-test (*p* < 0.05). Different letters in (**C**) indicate statistically significant differences based on two-way ANOVA and Tukey’s multiple comparisons test (*p* < 0.05).

To investigate the role of RsBPC5 in radish responses to Pb stress, transgenic hairy roots were generated by infecting radish seedlings with *Agrobacterium rhizogenes*. RT-qPCR analysis confirmed that *RsBPC5* expression in transient *OE-RsBPC5* plants was significantly higher than in the empty vector control (Figure 5A). Under Pb stress, transient *OE-RsBPC5* plants exhibited darker green, and well-expanded leaves compared to the empty vector control (Figure 5B). Chlorophyll content analysis revealed that, under Pb stress, transient *OE-RsBPC5* plants had higher levels of chlorophyll content (Figure 5C). These results suggest that RsBPC5 positively regulates radish tolerance to Pb stress.

### 2.7. Transient Overexpression of RsBPC5 Decreases the Pb Accumulation in Roots of R. sativus

**Figure 6:**
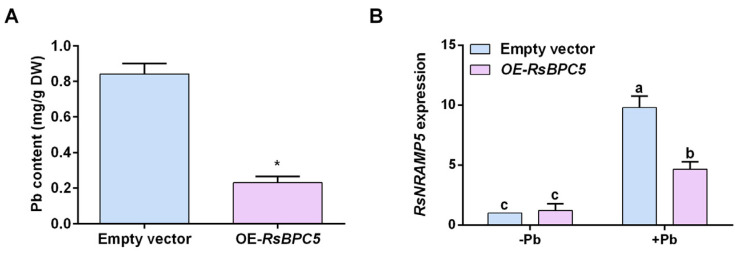
Effect of transient *RsBPC5* overexpression on Pb accumulation of roots in *R. sativus*. (**A**) Analysis of Pb content in transient *OE-RsBPC5* and empty vector roots after 6 days of Pb stress. (**B**) Analysis of *RsNRAMP5* expression in transient *OE-RsBPC5* after 6 days of Pb stress. NRAMP, natural resistance-associated macrophage protein. Experiments in (**A**,**B**) were performed in three independent biological replicates, and error bars represent the standard deviation (SD) calculated from these replicates. Asterisk in (**A**) indicates statistically significant differences as determined by Student’s *t*-test (*p* < 0.05). Different letters in (**B**) indicate statistically significant differences based on two-way ANOVA and Tukey’s multiple comparisons test (*p* < 0.05).

To further elucidate the function of RsBPC5 in modulating Pb stress responses, we measured Pb content in the roots of *OE-RsBPC5* and empty vector *R. sativus* seedlings. As shown in Figure 6A, Pb accumulation in *OE-RsBPC5* seedlings was obviously lower than that in the control group. This finding indicates that transient overexpression of *RsBPC5* plays a key role in affecting Pb contents. Moreover, the expression level of *RsNRAMP5*, a key metal transporter involved in Pb uptake, was higher in the empty vector group under Pb stress (Figure 6B). Collectively, these findings suggest that RsBPC5 negatively regulates the Pb accumulation in *R. sativus*, possibly by modulating the expression of *RsNRAMP5.*

### 2.8. Transient Overexpression of RsBPC5 Enhances the Antioxidant Activity in Roots of R. sativus

**Figure 7:**
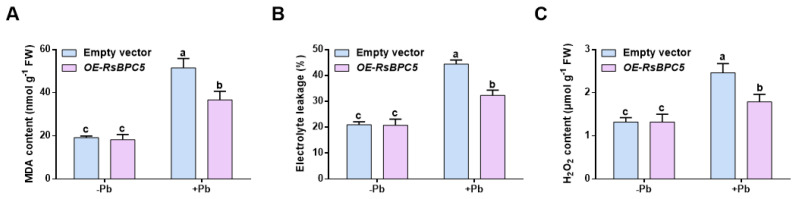
Effect of transient *RsBPC5* overexpression on oxidative damage in *R. sativus* roots under Pb treatment. MDA content (**A**), electrolyte leakage rate (**B**) and H_2_O_2_ content (**C**) in roots of *OE-RsBPC5* and empty vector seedlings after 6 days of Pb stress. Experiments in (**A**–**C**) were performed in three independent biological replicates, and error bars represent the standard deviation (SD) calculated from these replicates. Different letters in (**A**–**C**) indicate statistically significant differences based on two-way ANOVA and Tukey’s multiple comparisons test (*p* < 0.05).

In addition to limiting lead accumulation, lead stress stimulates the activation of antioxidant defense systems, which help mitigate oxidative damage caused by ROS. To assess this response, we first examined the extent of oxidative damage under lead stress. As expected, under lead exposure, the levels of malondialdehyde (MDA), hydrogen peroxide (H_2_O_2_), and electrolyte leakage in *OE-RsBPC5* roots were significantly lower than in empty vector seedlings (Figure 7).

**Figure 8:**
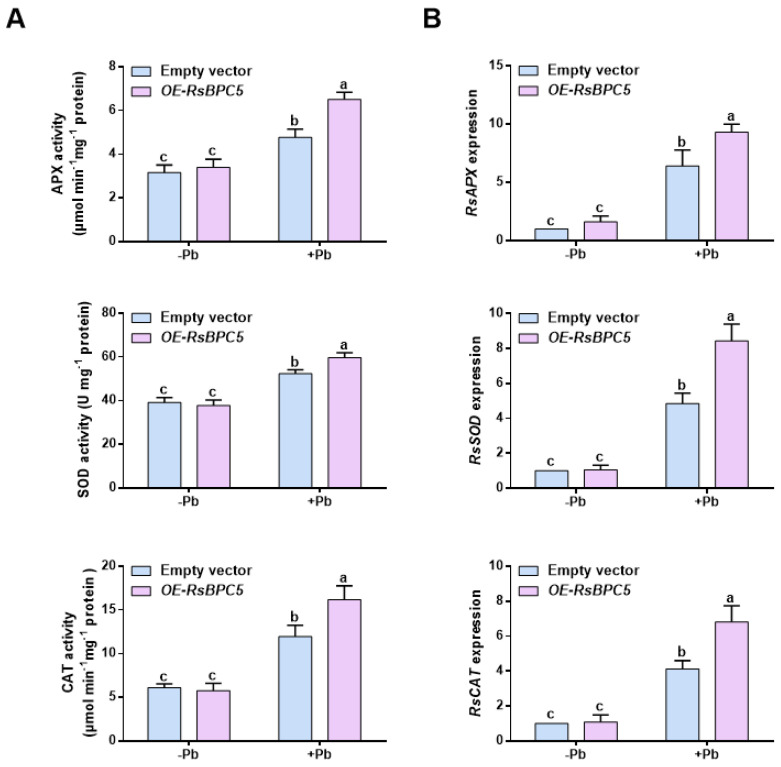
Effect of transient *RsBPC5* overexpression on the expressions and activities of antioxidant enzymes under Pb treatment. (**A**) The activities of superoxide dismutase (SOD), ascorbate peroxidase (APX), and catalase (CAT) enzymes in roots of *OE-RsBPC5* and empty vector seedlings after 6 days of Pb stress. (**B**) The relative expression of *RsSOD*, *RsAPX*, and *RsCAT* in roots of *OE-RsBPC5* and empty vector seedlings after 6 days of Pb stress. Experiments in (**A**,**B**) were performed in three independent biological replicates, and error bars represent the standard deviation (SD) calculated from these replicates. Different letters in (**A**,**B**) indicate statistically significant differences based on two-way ANOVA and Tukey’s multiple comparisons test (*p* < 0.05).

We next measured the activities of key antioxidant enzymes and found that, under lead stress, CAT, SOD, and APX activities were significantly higher in *OE-RsBPC5* roots compared to the empty vector controls (Figure 8A). These results suggest that the antioxidant defense response in *OE-RsBPC5* plants is enhanced to alleviate oxidative damage caused by Pb toxicity. Additionally, the transcript levels of key antioxidant genes (*RsAPX*, *RsSOD*, and *RsCAT*) were upregulated in *OE-RsBPC5* roots (Figure 8B), indicating active engagement of the antioxidant system in mitigating Pb-induced oxidative stress.

**Figure 9:**
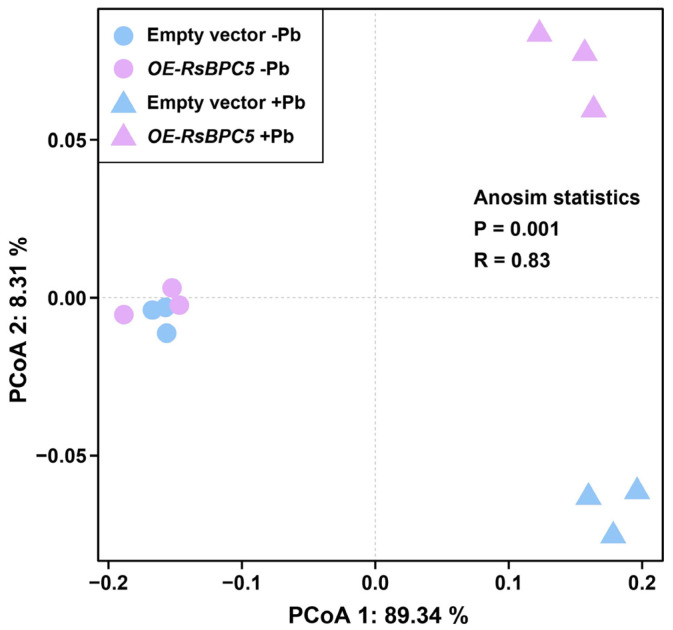
Principal coordinates analysis (PCoA) of radish physiological and transcriptional responses under Pb stress.

To further evaluate the global response of radish to Pb stress, a principal coordinates analysis (PCoA) was performed using integrated physiological and gene expression data (Figure 9). Under control conditions (–Pb), both *OE-RsBPC5* and empty vector groups clustered closely together, indicating comparable baseline physiological and transcriptional profiles. However, under Pb treatment (+Pb), the two groups exhibited clear separation in the PCoA plot, with *OE-RsBPC5* samples distinctly diverging from the control group. This pattern suggests that overexpression of *RsBPC5* markedly altered the overall response to Pb stress. The significance of this divergence was further supported by ANOSIM analysis (R = 0.83, *p* = 0.001), confirming that *RsBPC5* overexpression substantially reshapes the physiological and transcriptomic landscape of radish under Pb exposure.

## 3. Discussion

Transcription factors (TFs), such as NAC and WRKY, play critical roles in mediating plant responses to heavy metal stresses, including Pb toxicity [29]. These TFs regulate stress-related genes that enable plants to adapt to adverse environmental conditions. For example, in poplar, WRKY transcription factors were shown to enhance Pb tolerance [8]. In *Arabidopsis*, knockout of *AtbZIP20* or *AtbZIP47* increases sensitivity to Pb stress [9]. Although the roles of these TF families in Pb stress responses are well established, the function of the BASIC PENTACYSTEINE (BPC) family, primarily known for its involvement in developmental regulation, remains largely unexplored.

Previous studies have examined the expression pattern and function of *BPCs* in different plant species. Comparative genomic analyses have revealed species-specific variation in BPC number, with seven members identified in Arabidopsis thaliana. However, their characterization in radish (*Raphanus sativus*) remains unclear. Here, we identified 10 *BPC* genes (*RsBPCs*) in radish (Figure 1). The phylogenetic analysis revealed that the *BPC* genes in radish primarily cluster within Cluster III. This clustering suggests that these genes share evolutionary similarity and likely originated from a common ancestral gene. This pattern may indicate that radish has undergone an additional whole-genome triplication (WGT) event [30]. Among the RsBPCs, *RsBPC5* showed consistent downregulation under Pb stress, indicating its potential role in regulating Pb stress responses in radish.

Here, our study found that transient overexpression of *RsBPC5* significantly enhanced Pb tolerance compared to wild-type plants (Figure 5), and the RsBPC5 overexpression radish accumulated less Pb, suggesting a role for RsBPC5 in regulating Pb accumulation in roots. The absorption, accumulation, and translocation of cadmium in plants depend on the coordinated action of multiple heavy metal transporters [31,32]. Among them, the natural resistance-associated macrophage protein (NRAMP) family has received considerable attention due to its ability to transport a variety of trace elements, including zinc (Zn), iron (Fe), and Pb [33,34]. For instance, expression of *OsNRAMP5* in yeast significantly enhances lead uptake, whereas mutation of *OsNRAMP5* markedly decreases lead accumulation in the roots and grains of rice [35]. Notably, recent studies have investigated RsNRAMP5 in radish. Under lead stress, the transcription level of *RsNRAMP5* was significantly upregulated. Furthermore, overexpression of *RsNRAMP5* substantially inhibited yeast cell growth exposed to Pb stress [36]. Our findings further suggest that the transcription factor BPC may negatively regulate *RsNRAMP5* expression and contribute to this regulatory pathway. However, the observed downregulation of *RsBPC5* under Pb stress appears paradoxical if it indeed plays a positive role in Pb tolerance. One possible explanation is negative feedback regulation, whereby *RsBPC5* expression is suppressed following the activation of downstream stress responses to prevent excessive or prolonged signaling, which may be energetically costly or detrimental to the plant. Another possibility involves post-transcriptional regulation: the activity of RsBPC5 might be modulated at the protein level—such as through phosphorylation or interactions with other regulatory factors—allowing it to maintain its function even when transcript levels are reduced.

Additionally, plants initiate antioxidant defense mechanisms to counteract Pb toxicity. The primary defense limits Pb uptake into cells, while the secondary defense involves activating antioxidant systems to eliminate Pb-induced ROS. This defense strategy includes the induction of enzymatic antioxidants such as APX, SOD, CAT, and glutathione reductase, as well as non-enzymatic antioxidants including ascorbic acid, glutathione, carotenoids, and tocopherol. These antioxidants are distributed throughout cells and function in maintaining redox homeostasis [37]. For example, in maize, Pb stress induces physiological damage such as decreased chlorophyll content, increased proline accumulation, and elevated cell membrane permeability. To counteract these effects, maize plants enhance the antioxidant defense system, thereby reducing the extent of physiological damage [38]. Similarly, in soybeans, Pb stress significantly inhibits root and shoot growth and limits plant height development. Under such conditions, the plants alleviate the detrimental effects of Pb toxicity by increasing the activity of CAT and POD [39]. Previous transcriptome analysis revealed that genes involved in glutathione metabolism, such as *RsGST3-1*, may play important roles in the response of radish roots to Pb stress [40]. Our findings showed that RsBPC5 modulates ROS levels by regulating the activities of SOD, APX, and CAT. Furthermore, the expression levels of several antioxidant enzymes-related genes, such as *RsCAT*, *RsAPX* and *RsSOD*, in RsBPC5 overexpression radish were much higher when exposed to Pb stress (Figure 7 and Figure 8). These findings indicate that RsBPC5 enhances the antioxidant defense response in radish under lead stress by modulating ROS homeostasis through the regulation of both antioxidant enzyme activities and the expression of associated genes. It is worth noting that the increased enzymatic activities observed in this study are most likely a consequence of elevated enzyme abundance due to higher transcript levels, rather than changes in the specific activity of each enzyme molecule. Although we cannot exclude the possibility that RsBPC5 may also affect post-translational modifications or enzyme activation, such mechanisms were not directly examined in this study and require further investigation.

**Figure 10:**
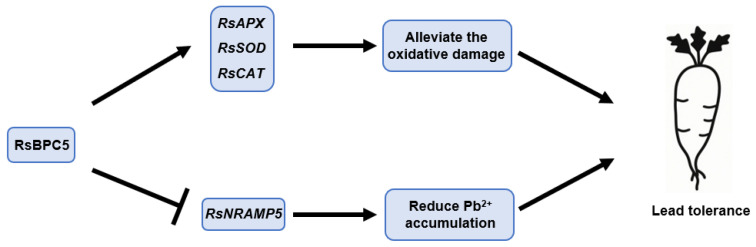
A proposed model of RsBPC5-mediated regulation of the Pb response in radish.

In conclusion, this study unveils a novel role for RsBPC5 in mediating plant responses to Pb stress and contributing to a better understanding of the transcriptional regulation underlying Pb tolerance (Figure 10). These findings contribute to our understanding of how plants adapt to heavy metal stress and offer potential strategies for developing Pb-tolerant crop varieties, supporting sustainable agricultural development. Notably, while our study primarily focused on Pb accumulation and the related molecular responses in Radish roots, it is important to acknowledge that Pb translocation to aerial tissues may also pose potential risks, particularly concerning food safety. Although the taproot is the main edible part of radish, leaves and stems are sometimes consumed or used as animal feed in certain regions. Therefore, future studies should comprehensively examine Pb distribution across different plant organs to provide a more complete assessment of the risks associated with Pb exposure through dietary intake.

## 4. Materials and Methods

### 4.1. Plant Materials

The 3-week-old radish seedlings were selected and hydroponically cultured in a 1/4-strength Hoagland and treated with 200 mg/L Pb (NO_3_)_2_, controls received ddH_2_O. Sampling was performed every two days, and the experiments were performed with three independent biological replicates. The culture chamber was maintained at 24 °C with a 14 h photoperiod and 20 °C with a 10 h dark period, under a relative humidity of 65%.

### 4.2. Characterization of BPCs in Radish

Amino acid sequences of all genes were extracted from the radish genome file and corresponding GFF annotation file [41]. As reference sequences, amino acid sequences of seven *Arabidopsis* BPCs (AtBPC1–AtBPC7) were obtained from the TAIR database and used as queries for BLAST alignment against the radish protein dataset, using a screening criterion of *p* < 1 × 10^−5^. Finally, 10 putative BPCs were identified in the radish genome through combined domain analysis using NCBI-CDD [42].

To analyze the structural features of BPC protein, conserved motifs were identified using the MEME Suite [43], and conserved domains were identified using the NCBI-CDD. Subsequently, the structure of BPC genes was determined using TBtools II software, based on the GFF3 annotation file of the radish genome [44].

For the phylogenetic analysis, BPC amino acids from *Amborella trichopoda*, *Oryza sativa* subsp. *japonica*, *Picea abies*, *Picea glauca*, *Zea mays*, and *Arabidopsis thaliana* were downloaded from the BBR-BPC Family dataset in PlantTFDB v5.0 [45]. These sequences were aligned with the 10 RsBPCs from *Raphanus sativus* using MAFFT Online. The aligned sequences were employed to construct a maximum likelihood (ML) phylogenetic tree using IQ-TREE with default parameters and 1000 bootstrap replicates. The generated phylogenetic tree was visualized and optimized using Evolview v2 [46].

### 4.3. Real-Time RT-qPCR Analysis

Total RNA of radish roots was extracted and reverse-transcribed into complementary DNA (cDNA) using the HiScript III RT SuperMix for qPCR (Vazyme, Nanjing, China) as described in [47]. Quantitative real-time PCR (RT-qPCR) was then conducted on a CFX96 Touch system (Bio-Rad Laboratories, Berkeley, CA, USA). Gene expression levels of *RsBPCs* were calculated using the 2^−ΔΔCT^ approach, with *RsActin* gene serving as the internal reference [48].

### 4.4. Subcellular Localization

The full-length CDS of *RsBPC5* was inserted into the pCAMBIA1300-GFP vector. The resulting RsBPC5-GFP fusion conduct was transformed into the *Agrobacterium tumefaciens* (GV3101), as previously described in [49]. Recombinant agrobacteria were infiltrated into 4-week-old *Nicotiana benthamiana* leaves. After a 72 h incubation, the infiltrated leaf tissues were examined with a confocal fluorescence microscope (excitation: 488 nm; emission: 527 nm).

### 4.5. Transcriptional Activity of RsBPC5

To evaluate the transcriptional activity of RsBPC5 in yeast, the full-length CDS of *RsBPC5* was inserted into the pGBKT7, which was transformed into the Y2HGold yeast strain. The Y2HGold cells transformed with RsBPC5 were cultured on a synthetic dropout/-Trp-His-Ade/X-α-gal (40 μg/mL) plate at 30 °C for 72 h.

To evaluate transcription activity of RsBPC5 in tobacco, the *RsBPC5* CDS was inserted into the pC1305-DBD vector and infiltrated into the tobacco leaves as described in [50]. Following 5 days of incubation, the LUC/REN ratio was quantified as an indicator of *RsBPC5* transcriptional activity.

### 4.6. Transformation of Hairy Roots in R. sativus

The recombinant pC1300 vector carrying the target gene was transformed into *Agrobacterium rhizogenes* MSU440 and cultured to an OD_600_ of 0.6. Bacterial cells were harvested and resuspended in 1/2 MS medium. Rootless radish seedlings were submerged in a bacterial inoculum for 10 min. With orbital shaking at 220 rpm, the infection was carried out at 28 °C. Following infection, seedlings were subjected to 2 days of dark co-cultivation, then transferred to a selective medium containing 500 mg/L cefotaxime and maintained under a 16 h light/8 h dark cycle at 25 °C.

### 4.7. Determination of Chlorophyll Content in Leaves of R. sativus

Chlorophyll content was determined using commercial assay kit (CPL-1-G, Cominbio, Suzhou, China). Briefly, 0.1 g fresh radish leaf tissue was homogenized with distilled water and Reagent 1, then transferred to a 10 mL glass tube. Rinse the mortar with pre-prepared extract solution (240 mL distilled water and 960 mL acetone), fill the tube to 10 mL, and incubate in darkness until decolorized. Finally, measure absorbance at 663 nm and 645 nm.

### 4.8. Determination of Oxidative Damage Index

MDA content was measured with an MDA assay kit (MDA-1-Y, Cominbio, China) according to the protocol in [51]. In brief, 0.1 g of thoroughly ground tissue was homogenized with 1 mL of extraction solution, and after centrifugation, the supernatant was mixed with 0.3 mL of reagent 1. The absorbance was then measured at 532 nm and 600 nm to calculate the ∆A for MDA content.

H_2_O_2_ levels were measured using an H_2_O_2_ assay kit (H_2_O_2_-1-Y, Cominbio, China) [52]. In brief, 0.1 g of tissue was homogenized with 1 mL of reagent 1, centrifuged, and the supernatant was mixed with reagents 1–3, followed by a second centrifugation. The resulting precipitate was dissolved with reagent 4, and absorbance was measured at 415 nm to calculate the H_2_O_2_ content.

Electrolyte leakage was assessed by incubating 0.1 g of fresh tissue in 40 mL of deionized water at 30 °C for 2 h in a 50 mL tube. After recording the electrical conductivity (EC1), the samples were boiled at 100 °C for 15 min to fully release intracellular ions. After cooling, final conductivity (EC2) was measured, and electrolyte leakage was calculated.

### 4.9. Determination of the Activities of Antioxidant Enzymes

APX activity was determined using the APX assay kit (APX-1-W, Cominbio, Suzhou, China). A crude APX extract was prepared from radish roots. After adding reagent I and reagent II, the absorbance at 290 nm was measured at 10 s and 130 s, and APX activity was calculated accordingly. CAT activity was measured using a CAT assay kit (CAT-1-Y, Cominbio, Suzhou, China). A crude extract of catalase from radish roots was prepared. The absorbance at 240 nm of each well was determined using a quartz microplate reader. One unit of CAT activity was defined as the amount of catalase that caused a decrease in A240 of 0.1 per minute at room temperature. The CAT activity per unit protein in the samples was calculated. SOD activity was measured using a SOD assay kit (TE0726, Leagene, Beijing, China). The extract was mixed with NBT working solution and then exposed to a 4000 Lx daylight lamp for 20 min. A dark reaction served as the blank control. Following reaction, the 560 nm absorbance was recorded on a microplate reader and converted to SOD activity per microgram protein.

### 4.10. Determination of Pb Content

Radish roots were dried to constant weight, ground, and sieved. Samples were weighed into digestion tubes and treated with 4 mL of 65% HNO_3_ and 1 mL of 30% H_2_O_2_. Microwave digestion was performed in three stages: 150 °C (40% power) for 15 min, 180 °C (60% power) for 15 min, and 100 °C (40% power) for 10 min. The Pb concentration was determined using an iCAP 7000 Plus ICP-OES (Thermo Scientific, Santa Clara, CA, USA) as described in [1].

### 4.11. Principal Coordinates Analysis

Principal coordinates analysis (PCoA) analysis was conducted based on combined genetic expression and physiological data using the Bray–Curtis distance metric. Analysis of similarities (ANOSIM) was further performed using the ‘vegan’ package in R (v4.4.1) to statistically test differences between treatment groups. Visualization of clustering patterns was also generated in R to illustrate sample-level distinctions.

### 4.12. Statistical Analysis

Data are presented as mean ± standard deviation (SD) based on three independent replicates. One-way or two-way ANOVA was performed using SPSS Statistics 20.0, and differences were deemed statistically significant at *p* < 0.05, while asterisks denote significant differences as determined by Student’s *t*-test (*p* < 0.05).

## Data Availability

The original contributions presented in this study are included in the article. Further inquiries can be directed to the corresponding author.

## Supplementary Materials

The following supporting information can be downloaded at: https://www.mdpi.com/article/10.3390/plants14152362/s1. Table S1: The ID number of BPC genes in various plant species; Table S2: Primers used in this study.
